# Expanded Access Programme: looking for a common definition

**DOI:** 10.1186/s13063-015-1108-0

**Published:** 2016-01-12

**Authors:** Antonella Iudicello, Lucia Alberghini, Giulia Benini, Paola Mosconi

**Affiliations:** Pharmaceutical Department, Azienda USL of Modena, Policlinico Hospital, Modena, Italy; Pharmaceutical Department, Azienda USL of Bologna, Maggiore Hospital, Largo B. Nigrisoli 2, 40133 Bologna, Italy; IRCCS Istituto di Ricerche Farmacologiche Mario Negri, Milano, Italy

**Keywords:** Expanded access programme, Compassionate use programme, Open-label extension study, Early access programme, Clinical trial

## Abstract

Therapeutic use of an unauthorised drug (or of an authorised drug for an unauthorised indication) for patients with a life-threating disease is permitted outside a clinical trial as an Expanded Access Programme (EAP).

The regulations regarding EAPs is not the same all over the world. For example, the recommendation of the European Medicines Agency (EMA) in EU countries also includes within EAPs patients who have been treated in a clinical trial and who wish to continue the treatment. Nevertheless, the patients treated in a clinical trial could have the option of continuing treatment for an extended period in an Open-label Extension study, aimed to generate long-term data on efficacy, safety, tolerability and administration.

The aims of this paper – based on the difficulties and incoherence encountered by an Italian Ethic Committee (EC) during the authorisation process of EAPs – are: understanding the origin of this misclassification by analysing differences and similarities among USA, European and Italian regulations concerning EAPs; and showing difficulties in classifying international study protocols as a consequence of the lack of harmonisation of definitions.

We performed a critical review of the current USA, European and Italian regulations and we analysed some practical cases by retrieving protocols from Clinicaltrials.gov and the Italian Clinical Trials Registry (OsSC) containing in the title the keywords ‘Expanded Access Programme’, “’Expanded Access’, ‘Open-label Extension study’ or ‘Early Access’.

We observed that the Food and Drug Administration ( FDA) definition of EAP is very clear while the EMA definition is similar to that of an Open-label Extension study. This lack of a clear definition generates misclassification and it is possible to find an EAP with an efficacy or safety endpoint; or an EAP managed as a clinical trial; or an EAP classified in Clinical Trials Registries as a phase II, III or IV clinical trial.

The internationalisation of the studies requires a harmonisation on a global level of legislation and definitions to eliminate misclassification of protocols. For this reason, the authors suggest that: a) the EMA definition should be harmonised with the FDA definition of EAPs, b) European regulation, even if optional, should be adopted in a compulsory way by national regulations. Moreover, separate registries for both EAPs and clinical trials should be organised.

## Background

Drugs are generally used according to their authorised indications by the competent authorities. However, there are situations where the use of an unauthorised drug, or intentional use of an authorised drug for a medical purpose not in accordance with the authorised product information (*off-label* use), is regulated and permitted, in clinical trials as well as in clinical practice. In a clinical trial, patients with therapeutic alternatives may decide whether or not to be treated with a drug whose safety and efficacy is still uncertain. In clinical practice, the use of an *off-label* drug is allowed when a patient does not have therapeutic alternatives.

If patients have a life-threating disease or condition, the therapeutic use of an unauthorised drug (or of an authorised drug for an unauthorised indication) is permitted outside a clinical trial in an Expanded Access Programme (EAP).

According to the Code of Federal Regulation (CFR) of the Food and Drug Administration (FDA) EAPs, sometimes also called Compassionate Use Programmes (CUPs), are proposed when manufacturers make an investigational drug available for therapeutic use, outside a clinical trial, to treat patients with a serious disease in the absence of comparable or satisfactory alternative therapeutic *on-label* drugs that cannot either participate or have already participated in a clinical trial [1].

This definition of EAP is not the same all over the world. For example, the recommendation of the European Medicines Agency (EMA) is for EU countries to include within EAPs patients who have been treated in a clinical trial and who wish to continue the treatment [2]. However, it is recognised worldwide that patients treated in a clinical trial could have the option of continuing treatment for an extended period in an Open-label Extension study in order to generate long-term data on the intervention efficacy, safety, tolerability and administration of the drug [3, 4]. Furthermore, in contrast with the FDA, in Europe CUPs and EAPs do not have the same meaning [1, 5].

The lack of a single shared definition, aims and common rules of the study protocols has already been reported by the European Clinical Research Infrastructures Network (ECRIN) [6]. A comprehensive survey on clinical research regulatory requirements showed that 10 European countries, covering approximately 70 % of the EU population, have adopted different requirements and that ‘Compassionate Use’ is not managed in the same way across Europe. For example, four of the ten countries included in the study have no formal regulatory system for this programme.

Today, international EAPs include patients from different countries where the legal framework and definitions of EAP are not the same. This calls for harmonisation, on a global level, of legislation and definitions.

This paper is the result of difficulties and incoherence encountered by an Italian Ethic Committee (EC) during the authorisation process of EAPs [1, 2] to recognise the coherence between the definition, aim and primary endpoint of study protocols presented as EAPs. This has implications, for example, on the way the study is conducted or also on the correctness of the information to be provided to the patients.

To understand the causes and underline the consequences of this incoherence, we have reviewed the current USA, European and Italian regulations and we have provided some case study examples.

## Review

### Methods

USA, European and Italian regulations and regulatory procedures concerning EAPs were retrieved, read and compared independently by two authors (IA, AL).

In August 2012, IA and AL retrieved and independently reviewed all studies with titles containing the keywords: ‘Expanded Access’, ‘Expanded Access Programme’, ‘Open-label Extension study’ and ‘Early Access’ from the Italian Clinical Trials Registry (OsSC) [7]. Only the studies recorded from January 2004 to June 2012 were considered because until 2004 there was no OsSC registry and it has been provisionally closed since June 2012.

The term ‘Compassionate Use Programme’ was not in the review because in the retrieved studies we did not reveal incoherence between the aim and endpoints of these programmes. In contrast with the FDA, in Europe CUPs and EAPs do not have the same meaning [1, 5].

The European EAPs have coherence problems between aim and endpoint, because for EMA legislation, adopted by Italian law, patients who have been treated in a clinical trial and who wish to continue the treatment can be included in an EAP [2]. As a consequence, sometimes European EAPs have the aims and endpoints of Open-label Extension studies.

For the eligible studies we tabulated the following details: NCT code, EudraCT number, trial code, title, design and number of arms, phase, investigational drug, therapeutic area, purpose, starting date, national/international trial, primary and secondary endpoints and sponsor. We also collected information about primary and secondary endpoints and verified their coherence with the aim of study protocol. The studies identified were then searched for in the American Clinical Trials Registry [8] and extracted when available. NCT code, EudraCT number, trial code and title were used to be sure that identified protocols were the same.

We classified the studies in the following clusters:

- EAPs according to the FDA (Code of Federal Regulation) [9], such as studies designed to ensure that potentially life-saving investigational drugs are available outside a clinical trial to patients with serious or immediately life-threatening diseases for which there is no comparable or satisfactory alternative treatment options, or to patients who either cannot participate or have already participated in a clinical trial: e.g. those who are not eligible because they have a different disease or stage of disease or otherwise do not meet the enrolment criteria, or because enrolment in the trial is closed, or because the trial site is not geographically accessible, etc. The cluster of EAPs included studies containing in the title the terms Expanded Access and Expanded Access Programme and Early Access
- Open-label Extension studies, according to the definition of Chin and Taylor [3, 4], such as studies following phase IIB or IIIA double-blind randomised placebo-controlled trials where the participant has the option of remaining in the study in an open-label fashion (i.e. they know which drug is being used) for an extended period of time (sometimes several years, often until the drug is licensed). Patients may be informed of this opportunity before or after the double-blind trial ends. Such studies generate long-term data on the efficacy, safety, tolerability and administration of the drug
- ‘Other’: studies that could not be univocally included in the previous groups

We used the FDA definition of EAPs because it clearly distinguishes the EAP from an Open-label Extension study, whereas this is not clear at all in the European and Italian regulations.

Descriptive statistics, mainly proportions, were used to analyse all data collected. Data was collected and analysed using Microsoft Excel (Microsoft Inc., Redmond, WA, USA).

## Results

### A matter of classification

The USA, EMA and Italian EAP regulations are compared in Table 1.

**Table 1:** Expanded Access Programme: comparison of definition

USA – Food and Drug Administration	Europe – European Medicines Agency	Italy – Ministerial Decree
A case-by-case basis for individual patients, including for emergency use Under this section, FDA may permit an investigational drug to be used for the treatment of an individual patient by a licensed physician. The following characteristics must be respected: (1) The physician must determine that the probable risk to the person from the investigational drug is not greater than the probable risk from the disease or condition (2) FDA must determine that the patient cannot obtain the drug under another Investigational New Drug (IND) application or protocol (3) Treatment is generally limited to a single course of therapy for a specified duration unless FDA expressly authorises multiple courses or chronic therapy (4) At the conclusion of treatment, the licensed physician or sponsor must provide FDA with a written summary of the results of the Expanded Access use, including adverse effects (5) When a significant number of similar individual patient Expanded Access requests have been submitted, FDA may ask the sponsor to submit an IND or protocol for the use under an Expanded Access for an intermediate-size patient population or Expanded Access for larger group(s) of patients under a treatment protocol or treatment IND Moreover, the preamble to the proposed rule stated that to support Expanded Access for an individual patient when the patient has an immediately life-threatening condition that is not responsive to available therapy, ordinarily completed phase 1 safety testing in humans at doses similar to those to be used in the treatment, together with preliminary evidence suggesting possible effectiveness, would be sufficient to support such use. However, the preamble further stated that in some cases there may be no relevant clinical experience, and the case for the potential benefit may be based on preclinical data or on the mechanism of action	An individual patient (Compassionate Use on a Named Patient basis) The following characteristics must be respected: (1) A EU Member State (EuMS) may, in accordance with legislation in force and to fulfil special needs, authorise the use of a medicinal product, otherwise than for the authorised indications or the use of an unauthorised medicinal product, supplied in response to a bona fide unsolicited order, formulated by an authorised health-care professional for use by an individual patient under his direct personal responsibility (2) A EuMS may temporarily authorise the distribution of an unauthorised medicinal product in response to the suspected or confirmed spread of pathogenic agents, toxins, chemical agents or nuclear radiation, any of which could cause harm (3) The doctor responsible for the treatment will contact the manufacturer directly	In Italy, the therapeutic use of drugs undergoing clinical trials outside a clinical trial is permitted when there is no comparable or satisfactory therapeutic alternative for a severe pathology or rare disease or condition that could be life-threatening The following characteristics must be respected: (1) The drug is already the subject of ongoing or concluded phase III clinical trials for the same specific therapeutic indication (2) In specific cases of life-threatening conditions , the drug is the subject of concluded phase II clinical trials. The drug can be obtained by: a. physician for an individual patient not treated in a clinical trial; b. several physicians working in different centres or collaborative multicentre groups; c. physicians or collaborative groups, for patients who have participated in a clinical trial which has shown effectiveness and tolerability profiles that make it useful, for those who have participate in the trial to exploit the results promptly
Intermediate-size patient population Under this section, FDA may permit an investigational drug to be used for the treatment of a patient population (smaller than those typical of a treatment IND or treatment protocol: 1–100 patients) with similar treatment needs who otherwise do not qualify to participate in a clinical trial. FDA may ask a sponsor to consolidate Expanded Access under this section when the agency has received a significant number of requests for individual patient Expanded Access to an investigational drug for the same use. The following characteristics must be respected: (1) There is enough evidence that the drug is safe at the dose and duration proposed for Expanded Access use to justify a clinical trial of the drug in the approximate number of patients expected to receive the drug under Expanded Access (2) There is at least enough preliminary clinical evidence of effectiveness of the drug, or of a plausible pharmacologic effect of the drug, to make Expanded Access use a reasonable therapeutic option in the expected patient population (3) If the number of patients enrolled increases, FDA may ask the sponsor to submit an IND or a treatment protocol Expanded Access under this section may be needed in the following situations: a) The drug is not being developed: for example, because the disease or condition is so rare that the sponsor is unable to recruit patients for a clinical trial.b) The drug is being studied in a clinical trial, but patients are unable to participate in the trial because they have a different disease or stage of disease or otherwise do not meet the enrolment criteria; because enrolment in the trial is closed, or because the trial site is not geographically accessible. c) The drug is an approved product that is no longer marketed for safety reasons or is unavailable on the market due to failure to meet the conditions of the approved application; d) The drug contains the same active moiety as an approved product that is unavailable on the market due to failure to meet the conditions of the approved application or a drug shortage Moreover, the Expanded Access submission must state whether the drug is being developed or is not being developed and describe the patient population to be treated. If the drug is not being actively developed, the sponsor must explain why the drug cannot currently be developed for Expanded Access use and under what circumstances the drug could be developed. If the drug is being studied in a clinical trial, the sponsor must explain why the patients to be treated cannot be enrolled in the clinical trial and under what circumstances the sponsor will conduct a clinical trial in these patients.	Group of patients (Compassionate Use Programmes) According to Article 83, the aim of this group is: (1) To facilitate and improve the access of patients in the EU to Compassionate Use Programmes (2) To favour a common approach regarding the conditions of use, the conditions for distribution and the patients targeted for the compassionate use of unauthorised new medicinal products (3) To increase transparency between EuMSs in terms of treatment availability It is applicable to: a) Unauthorised medicinal products for human use falling within the categories referred to in Articles 3(1) and 3(2) of Regulation (EC) No. 726/2004; b) ‘'*Patients with a chronically or seriously debilitating disease, or a life-threatening disease, and who cannot be treated satisfactorily* ^*a*^ *by unauthorised medicinal product*’ in the EU, or who cannot enter a clinical trial; c) *Group of patients* ^b^; d) Medicinal product which is either ‘the subject of an application for a centralised marketing authorisation in accordance with Article 6 of Regulation (EC) No. 726/2004 or is undergoing clinical trials’ in the EU or elsewhere It not is applicable to: a) Use of an unauthorised medicinal product for Compassionate Use on a Named Patient basis (as meant in Article 5 of Directive 2001/83/EC); b) A medicinal product which has already been authorised via the Centralised Procedure, even if the proposed conditions of use and target population are different from those of the marketing authorisation; c) Medicinal products which are not eligible for the Centralised Procedure. Article 83 on compassionate use is complementary to national legislations and provides an option to MS who wish to receive a CHMP opinion regarding the conditions for compassionate use of a specific medicinal product which falls within the scope of Article 83.	
Large populations Under this section, FDA may FDA may permit an investigational drug to be used for widespread treatment use. The following characteristics must be respected: (1) The drug is being investigated in a controlled clinical trial under an IND designed to support a marketing application for Expanded Access use, or all clinical trials of the drug have been completed (2) When the safety and potential effectiveness of drug is known from ongoing or completed clinical trials (3) The sponsor is actively pursuing marketing approval of the drug for Expanded Access use, with due diligence (4) When Expanded Access use is for a serious disease or condition, there is sufficient clinical evidence (phase 3 or completed phase 2 studies) of safety and effectiveness to support Expanded Access use (5) When the Expanded Access use is for an immediately life-threatening disease or condition, the available scientific evidence (phase 3 or completed phase 2 studies, but could be based on more preliminary clinical evidence) provides a reasonable basis to conclude that the investigational drug may be effective and would not expose patients to an unreasonable and significant risk of illness or injury		
	Expanded Access Programmes (EAPs) A company that makes a promising medicine may choose to run an EAP to allow early access to their medicine and to widen its use to patients who can benefit from it. For example, patients who have been treated with the medicine during a clinical trial and wish to continue treatment may be able to do so via an EAP. These programmes are often authorised by national authorities in the same way as clinical trials, and patients are followed in the same way as in a clinical trial	

^a^’*Patients who cannot be treated satisfactorily*’ means patients left without treatment options or patients whose disease does not respond or relapses to available treatments, or for whom the treatments are contraindicated or inadequate [11]

^b^’*Group of patients*’ can be interpreted as any set (i.e. more than one) of individual patients that would benefit from a treatment for a specific condition. The terms ‘cohort’, ‘collective use’, ‘patient group prescription’ or ‘special treatment programme’ used in some MSs, in accordance with national legislations, may correspond with this concept [11]

*CHMP* Committee for Medicinal Products for Human Use, *EC* Ethic Committee, *EU* European Union, *FDA* Food and Drug Administration, *MS* member state

In the United States, the investigational drug is available either through a clinical trial or through an EAP. The EAP (sometimes also called CUP) is regulated by Title 21 of the Code of Federal Regulations [1]. For the FDA an EAP is a way to make potentially life-saving drugs available to patients in certain serious circumstances or with immediately life-threatening diseases for which there are no comparable or satisfactory alternative treatment options. Compared to the use of an investigational drug in the usual studies under an Investigational New Drug (IND) application, the EAP’s primary aim is not to obtain information about the safety or effectiveness of a drug [10]. The general criteria for an EAP are that: patient(s) to be treated suffer from a serious or immediately life-threatening disease for which there is no comparable or satisfactory alternative therapy; and the potential benefit justifies the potential risks of the use and are not unreasonable in relation to the disease to be treated [9].

There are three categories of EAP: for individual patients, for intermediate-size patient populations and for large populations of patients, who have no other treatment option available, under a treatment protocol or treatment IND application (see Table 1) [9].

In all categories of EAP the patients either cannot participate or have already participated in a clinical trial for the following reasons: patients have a different disease or stage of disease or otherwise do not meet with the enrolment criteria; enrolment in the trial is closed; and the trial site is not geographically accessible. The FDA establishes that early access to an investigational drug will not interfere with clinical trials, since that is the most effective and efficient way to establish whether the drug works and is safe to use.

In Europe, EAP and CUP do not have the same meaning. EMA identifies compassionate use to facilitate patients’ access to new treatment options under development for an individual patient as Compassionate Use on a Named Patient basis, or for a group of patients as CUPs [11].

The first is regulated by Article 5 of Directive 2001/83/EC [12] defining the conditions under which physicians can obtain, under their direct responsibility, an unauthorised drug or a drug otherwise than for the authorised indications for individual patients with no *on-label* therapeutic alternatives (see Table 2). The second is regulated by Article 83 of Regulation (EC) No. 726/2004 [13], which introduces the legal framework for the use of an unauthorised drug (but eligible for being authorised through the Centralised Procedure), for groups of patients with life-threatening, long-lasting or seriously disabling illnesses who currently cannot be treated satisfactorily with authorised medicines, or who have a disease for which no medicine has been authorised yet, or who are not eligible for an ongoing clinical trial to obtain treatment with a potentially life-saving medicine. The assumptions for compassionate use must be based on mature randomised phase III trials or exploratory trials (e.g. uncontrolled phase II trials). The scientific data submitted should permit assessment of the conditions for effective and safe use of the medicinal product in a CUP. The company should ensure that patients taking part in a CUP have access to the product during the period between the granting of the centralised marketing authorisation and its placement on the market [5].

**Table 2:** Examples of use on a named patient basis

✓ The licensed form of a drug has been discontinued can be prescribed on a named patient basis as it does not have a license
✓ The product is awaiting a license but is licensed elsewhere in the world
✓ There is a local shortage or supply chain problems for an essential medication
✓ Patients who have taken part in a clinical trial which has now ceased can still be prescribed the ‘trial’ medication on a named patient basis if it has been achieving good results
✓ There is a clinical need for an agent which is still in clinical trials and the patient is not eligible for the trial, but it is felt they may benefit from the drug
Specials prepared in a hospital pharmacy for individual patients, because these items do not exist in a ready-made form

The EMA permits companies that manufacture promising medicines to run CUPs to allow early access to their medicine and to extend its use to patients who can benefit from it. But EMA also permit that patients who have been treated with the medicine during a clinical trial and wish to continue using it may be able to do so via an EAP [2].

EMA and FDA compassionate use definitions have the same meaning. Conversely, the EMA definition of EAP is similar to that of an Open-label Extension study.

In general, the European Medicines Agency’s Committee for Medicinal Products for Human Use (CHMP) provides the recommendations to all European Member States (EuMS) on access to a medical product for unauthorised indications, outside a clinical trial. The recommendations complement national legislation, and do not replace it. They also do not create any legal framework in the EuMSs. The recommendations are optional, and are only implemented by EuMSs that wish to implement them for their patients [2, 5, 13].

In Italy, access to an unauthorised drug or an authorised drug not in accordance with the authorised product information has been regulated by various decrees (DLvo No. 648/1996, DLvo No. 94/1998, DLvo No. 211/2003, Ministerial Decree 8 May 2003) [14–17]. The latter Ministerial Decree defines the terms of access to an EAP (also called Early Access Programme) with experimental drugs, outside a clinical trial, when there is no comparable or satisfactory therapeutic alternative for severe pathology or a rare disease or illness that could be a life-threatening [14]. Access to such therapies is possible if at least one of the following conditions is met: the drug is already being tested in the same specific therapeutic indication in ongoing or concluded phase III or in concluded phase II clinical trials and the available data must be sufficient to allow a positive judgment on the effectiveness and the tolerability of the drug. The supply of the drug can be requested by: a) the physician for an individual patient not treated in a clinical trial; b) several physicians working in different centres, or collaborative multicentric groups; or c) physicians or collaborative groups, for patients who have already participated in a clinical trial with good effectiveness and tolerability profiles where the results must be used promptly. Italian law has adopted the EMA definition of EAP. Treatment data must be collected as for observational studies and can be used as ‘support’ of the marketing authorisation dossier [14].

Worldwide Open-label Extension studies, defined as clinical trials that follow a double-blind randomised placebo-controlled trial phase IIB or IIIA, aim to generate long-term data on the intervention efficacy, safety, tolerability and administration, and participants have the option of continuing treatment for an extended period [3].

### Results from clinical trial records

In the considered period, 172 protocols were identified through the OsSC:

- Twenty-one ‘EAPs’, of which 2 were for patients with an available therapeutic alternative
- One hundred and fifty ‘Open-label Extension studies’, of which 99 were coherent with study definition, 49 were core studies that included the extension in the study protocol and 2 were extensions of another extension
- One ‘Other’ because it was registered as Extension Study of EAP in the American Clinical Trials Registry, while in the OsSC it was registered as an Expanded Access Study

More than 50 % of the protocols concerned 3 therapeutic areas: nervous system diseases (30 %, 52/172), oncology (12 %, 21/172) and immune system diseases (12 %, 21/172). Almost all protocols (99 %) were multicentre with a median sample size of 780 patients, and 88 % (151/172) were international.

Focusing on the 21 EAPs, 52 % (11/21) had as their primary endpoint ‘to give the possibility of early use of the drug to patients who have no comparable or satisfactory therapeutic alternative and cannot participate in the clinical trial’, while 43 % (9/21) presented a primary endpoint of long-term safety and/or tolerability. Almost all of the EAPs (95 %, 20/21) were sponsored by pharmaceutical companies.

We encountered difficulties in classifying the study protocols because in some cases (16 of 21) protocols that have the purpose of providing the treatment to patients who are not eligible for enrolment into a clinical trials are presented as EAPs, in some cases (3 of 21) studies that follow a clinical trial and include patients who wish to continue the treatment are presented as EAPs, in others cases (2 of 21) clinical trials are presented as EAPs. We also found EAPs classified as phase II, III or IV clinical trials.

This is due to lack of a common definition of EAP: European and Italian regulations provide the option to include in an EAP patients who have been treated in a clinical trial and wish to continue treatment; however, for the FDA the EAP guarantees treatment to patients who cannot participate in a clinical trial, while participants in a clinical trial have the option to enter an Open-label Extension study, not an EAP.

Tables 3, 4 and 5 present 3 case-models showing the consequences of this lack of harmonisation. In all cases the EAPs are managed like clinical trials, they are registered in OsSC and/or Clinicaltrials.gov and/or other trial registries, but without following the rules for clinical trials; for example, the sponsor is not available to cover the costs of drugs in the add-on therapy of the experimental arm.

**Table 3:** CASE MODEL 1: Example of protocols of EAP that have the purpose of providing the treatment to patients who are not eligible for enrolment into a clinical trials.

Case model 1 Therapeutic area: Neoplasia				
Clinical study design	Target	Endpoints	Italian or international	Phase
Multicentre, open-label, single-arm, expanded-access	Advanced stage carcinoma (inoperable or metastatic)	Primary: assess the safety and tolerability of long-term treatment	Italy and other European countries	Phase 3
	Progressive disease after treatment with marketed drugs or intolerance	Secondary: assess progression-free and overall survival		
	Patients not previously treated with the experimental drug (who has not participated in a clinical trial)			

In this case-model, patients included in the protocol have no comparable or satisfactory therapeutic alternatives but the protocol has safety and tolerability primary endpoints

Considerations: this protocol (on the basis of the target), can come under Ministerial Decree 8 May 8 2003, as a CUP and EAP, because it ensures access to investigational drugs for patients who have no comparable or satisfactory therapeutic alternatives. However, the primary endpoint is typical of a clinical trial

**Table 4:** CASE MODEL 2: Example of protocols of EAP that follow a clinical trial and include patients who wish to continue the treatmentᅟ

Case model 2 Therapeutic area: Urogenital diseases				
Clinical study design	Target	Endpoints	Italian or international	Phase
Multicentre, open-label, single-arm, early-access	Patients who have participated in the clinical trial and need prompt access to the results	Primary: assess the safety and tolerability in long-term treatment	Italy and other European countries	Phase 3

In this case-model, patients included in the protocol have no comparable or satisfactory therapeutic alternatives but they have participated in the clinical trial and need to continue the treatment

Considerations: despite the protocol target being coherent in Italy and in Europe with the EAP, the primary endpoint is typical of a clinical trial and the sponsor manages the protocol as if it were a clinical trial. In the USA this protocol would have be configured as an Open-label Extension study

**Table 5:** CASE MODEL 3: Example of clinical trial who is presented as EAPsᅟ

Case model 3 Therapeutic area: Viral diseases				
Clinical study design	Target	Endpoints	Italian or international	Phase
Multicentre, open-label, single-arm, early-access	Patients with chronic hepatitis C, acute fibrosis and cirrhosis	Primary: make possible early use of the drug for patients who cannot participate in the clinical trial	International	Phase 3
	Patients with similar features to those in the clinical trial but unable to participate because the trial is closed or the trial site is not geographically accessible, or because not eligible under ongoing trial protocols	Secondary: assess the safety and tolerability of the treatment		

In this case-model, patients included in the protocol have no comparable or satisfactory therapeutic alternative and they cannot participate in the clinical trial

Considerations: since the purpose of the protocol is to make possible early use of the drug for patients who have no comparable or satisfactory therapeutic alternative and cannot participate in the clinical trial, this protocol can come under Ministerial Decree 8 May 2003 [14], as a CUP [11] and EAP [21]. However, the study is managed like a clinical trial, and the cost of the add-on therapy is charged to the centre where the trial takes place

## Discussion

This study shows how the definition of EAP is not the same all over the world. The comparison of the USA, European and Italian regulations highlights several similarities and differences.

Regarding the similarities:

- The patient must have a life-threatening, long-lasting or seriously disabling illness
- The patient has no comparable or satisfactory alternative *on-label* therapy
- The aim of an EAP is to simplify the therapeutic use of an unauthorised drug (or of an authorised drug for indication unauthorised) outside a clinical trial
- The data on clinical trials (and pre-clinical trial for FDA case-by-case) must be sufficient to permit a positive judgment on the efficacy and tolerability of the drug
- EAPs cannot be used for investigational purposes or commercial pre-authorisation activities
- EAPs regard individual patients or groups of patients

There are, then, some significant differences:

- FDA (a) defines as EAP (also called CUP) the access protocols for experimental pharmacological therapies outside a clinical trial; (b) these protocols are applied for individual patients, intermediate-size patient populations and large populations; and (c) the patients who can participate in clinical trials are excluded
- EMA (a) defines the access protocols to experimental pharmacological drugs outside a clinical trial as a CUP and Compassionate Use on a Named Patient basis; (b) these protocols are applied for groups of patients or individuals, respectively

EAPs represent for EMA a way to continue the treatment for a patient who has been treated with a drug during a clinical trial

- In Italy (a) the therapeutic use of an investigational drug outside a clinical trial is defined as an EAP (also called Early Access Programme); (b) the EAP involves both individuals and groups; and (c) patients who have already participated in a clinical trial and need to use the randomised controlled trial (RCT) results quickly are included

Therefore, in the USA the EAP has just a therapeutic endpoint, whereas for European and Italian regulations the EAP could also have efficacy and/or safety endpoints.

### EAP versus Open-label Extension studies

The FDA regulation clearly distinguishes EAP and Open-label Extension studies [1], whereas this is not clear at all for European and Italian regulations [2, 14]. For the latter, patients who have been treated with the investigational drug during a clinical trial can enter an EAP or an Open-label Extension study as well. However, in an Open-label Extension study all patients who have participated in the study core (experimental and control arm) have access to the experimental treatment. Instead, in an EAP only patients who have been treated with the investigational drug (not the control arm) during a clinical trial can continue treatment [18]. This lack of a clear distinction generates misunderstandings.

For European and Italian regulations, the studies defined as EAPs (also called Early Access Programmes) could have scientific endpoints as Open-label Extension studies. This is possible because EMA recommendations presuppose that safety data can also be collected during CUPs [12] and the Italian Ministerial Decree 8 May 2003 provides for the use of the data obtained from EAPs in ‘support’ of the marketing authorisation dossier [14]. FDA regulation, on the other hand, excludes the fact that the primary intention of Expanded Access uses is to obtain information about the safety or effectiveness of a drug, and also excludes the fact that the data obtained from EAPs can interfere with the authorisation procedure for a new drug [19].

Furthermore, in Italy for EAPs (also called Early Access Programmes) the sponsor is not open to covering the costs of standard care, any add-on therapy and managing it as an Open-label Extension study which imposes a part of the cost on the trial centre.

### EAP versus clinical trials

According to all examined regulations, wherever the physician directing the treatment starts an EAP he must sign up as the clinical investigator/s and submit to the EC and to the competent authority (e.g. AIFA in Italy) a study protocol including and adequately documenting the following:

- The rationale for the intended use of the drug
- The criteria for patient selection
- The available data on the quality, safety and efficacy of the medicinal product
- The scientific data submitted should allow an assessment of whether the drug is reasonably safe at the dose and duration proposed for the intended target population
- Patients who enter in an EAP must all sign an Informed Consent Form

These criteria are the same for phase II and III clinical trials and it is probably for this reason that sponsors may wrongly interpret EAPs as clinical trials, although 1) FDA and EMA point out: ‘*patients should always be considered for inclusion in a clinical trial before being offered programmes outside the clinical trial*’ [2]; 2) data collection on safety and efficacy is the primary objective of a clinical trial [20–23]; and 3) only clinical trials can be generally recognised as a ‘*scientifically valid and reliable*’ way to provide safety and efficacy data transferable on the general population. In other words, ‘*From a methodological point of view, clinical trials are practically the only means to obtain reliable and interpretable efficacy and safety data for a medicinal product*’ [4].

The problem is that according to European regulation, ‘*The use of drug outside to clinical trial is often authorised by national authorities in the same way as clinical trials, and patients are followed in the same way as patients in a clinical trial*’ [2, 24, 25].

Therefore, there should be a clear distinction between EAPs and clinical trials: a protocol designed to provide for an early access drug (therapeutic endpoint), when there is no comparable or satisfactory therapeutic alternative, should be clearly distinguished from a clinical trial whose aim is to assess the safety and efficacy of investigational drugs (scientific endpoint).

To clearly define ‘*the rules of the game*’, the intervention of regulatory agencies would be necessary. Normally, EAPs are conducted in parallel with the drug clinical trials or when the drug is already under assessment for marketing authorisation by a competent authority, or prior to its launch. As such, EAPs may be initiated as early as phases II/III, but can start at any time during the approval process or after being granted a market authorisation but prior to market launch, a period which may cover 1 to 2 years. As sufficient safety and efficiency data is needed for the approval of these programmes, application is more feasible late in the drug development. However, under exceptional circumstances, drugs may obtain early access with very little clinical information such as for Ebola or H1N1 pandemic risk.

## Conclusions

While the FDA definition of EAPs is very clear, the EMA definition of EAPs is similar to the definition of Open-label Extension studies. The internationalisation of the studies requires a harmonisation on a global level of legislation and correct/clear definition to eliminate misclassification of study protocols. As a consequences of misclassification, it is possible to find an EAP with an efficacy or safety endpoint or an EAP managed as a clinical trial or EAPs classified in the trial registries as phase II, III or IV clinical trials.

To avoid misclassification the authors suggest that: a) EMA definition should be harmonised starting from the FDA definition; and b) European regulation should not be optional but it should be adopted compulsorily by national regulations. Moreover, separate registries for both EAP and clinical trials could give researchers/clinicians and patients a better understanding of the situation in which they are receiving the drug, they are not in a clinical trial and they are not receiving a treatment expected ‘from normal clinical practice’. Separated registry could allow simplification of protocol assessment by the authorities and national ECs and it could also exclude the fact that efficacy data obtained from EAPs are used by the sponsors for registration purposes because data obtained from EAPs should not interfere [14, 20] with the authorisation procedure for a new drug but should increase information for pharmacovigilance plans.

To protect patients, European legislation needs to be more explicit and informative with regard to the regulatory requirements, restrictions, and responsibilities in EAPs. EAPs and clinical trials must be separated, firstly to protect patients from exploitation, and secondly so that both the needs of the seriously ill and those of society can best be served [6].

## Abbreviations

CFR: Code of Federal Regulation
CHMP: Committee for Medicinal Products for Human Use
CUP: Compassionate Use Programme
EAP: Expanded Access Programme
EC: Ethic Committee
ECRIN: European Clinical Research Infrastructures Network
EMA: European Medicine Agency
EuMS: European Member States
FDA: Food and Drug Administration
IND: Investigational New Drug
OsSC: Italian Clinical Trials Registry
RCT: Randomised controlled trial
